# Plasma D-Dimer Concentrations and Risk of Intracerebral Hemorrhage: A Systematic Review and Meta-Analysis

**DOI:** 10.3389/fneur.2018.01114

**Published:** 2018-12-20

**Authors:** Zhike Zhou, Yifan Liang, Xiaoqian Zhang, Junjie Xu, Kexin Kang, Huiling Qu, Chuansheng Zhao, Mei Zhao

**Affiliations:** ^1^Department of Geriatrics, The First Affiliated Hospital, China Medical University, Shenyang, China; ^2^Department of Neurology, The First Affiliated Hospital, China Medical University, Shenyang, China; ^3^Department of Laboratory Medicine, The First Affiliated Hospital, China Medical University, Shenyang, China; ^4^Department of Neurology, People's Hospital of Liaoning Province, Shenyang, China; ^5^Department of Cardiology, The Shengjing Affiliated Hospital, China Medical University, Shenyang, China

**Keywords:** plasma d-dimer, intracerebral hemorrhage, meta-analysis, risk factor, arteriovenous malformation

## Abstract

**Background:** The aim of our meta-analysis was to evaluate the association between plasma d-dimer and intracerebral hemorrhage (ICH).

**Methods:** Embase, Pubmed, and Web of Science were searched up to the date of March 19th, 2018, and manual searching was used to extract additional articles. Standard mean difference (SMD) with 95% confidence intervals (CI) was calculated to evaluate d-dimer levels.

**Results:** Thirteen studies including 891 ICH patients and 1,573 healthy controls were included. Our results revealed that higher levels of d-dimer were displayed in ICH patients than those in healthy controls (95% CI= 0.98–2.00, *p*< 0.001). Subgroup analysis based on continent of Asia and Europe, sample size, as well as age in relation to d-dimer levels between ICH patients and healthy controls did not change the initial observation; whereas no differences of d-dimer levels were found between ICH and controls in America.

**Conclusions:** This meta-analysis revealed that high level of d-dimer is associated with the risk of ICH. Plasma d-dimer is suggested to be a potential biomarker for patients with ICH in Asia and Europe rather than in America. There were no impact of sample size-related differences and age-related diversities on the risk of ICH with respect to d-dimer levels.

## Introduction

Intracerebral hemorrhage (ICH), also known as cerebral bleed, is described as spontaneous extravasation of blood into the brain parenchyma (1). This clinical entity accounting for ~20–30% of all strokes in Asia and 10–15% of strokes in the USA, Australia and Europe, is generally considered the most lethal subtype of stroke with high morbidity and mortality (2–4). ICH is a multifactorial disease whose etiology has not been fully elucidated. In addition to conventional risk factors mainly hypertension combined with arteriosclerosis, cerebral arteriovenous malformation (AVM), cerebral aneurysm and cerebral amyloid angiopathy, there is evidence that hemostatic condition undergoes detailed changes in patients with ICH, implying that the coagulopathy may have some underlying mechanism linking the pathogenesis of ICH (5).

D-dimer, a fibrinogen compound with high molecular weight, is formed during activation of the coagulation system and derived from the degradation of cross-linked fibrinogen (6). *In vivo*, the activation of the thrombosis and fibrination could be reflected by the appearance and elevation of plasma d-dimer, which appeared to be one of the most valuable evaluation parameters for the investigation of thrombolysis (7, 8). There is convincing evidence that the level of d-dimer is higher in ischemic stroke with large infarction area and high disability scores (9). However, whether elevated level of plasma d-dimer has been related to ICH or not remains unconfirmed. Therefore, it is worthwhile to conduct a systematic review and meta-analysis to evaluate the relationship of plasma d-dimer concentrations in relation to the risk of ICH.

## Materials and Methods

### Inclusion and Exclusion Criteria

Studies were considered eligible if they met the following criteria: (a) case-control studies or retrospective studies; (b) the case group was defined as patients who had an acute ICH based on the World Health Organization criteria and those with subarachnoid hemorrhage, coagulation diseases, liver impairment, heart failure, and malignant disease were excluded; the control group was defined as age- and sex-matched healthy subjects having no liver dysfunction, no diagnosis of heart disease, and no history of antiplatelet or anticoagulation therapy. (c) enough data was provided to investigate the relationship between plasma d-dimer and ICH patients; (d) blood samples were collected at baseline or after admission to assess the level of d-dimer; (e) d-dimer level was reported in the ICH patients and healthy controls. Exclusion criteria were as follow: (a) not conforming to the inclusion criteria; (b) duplicated publications or studies with overlapping data; (c) abstracts, proceedings, letters, reviews, meta-analysis, or case reports.

### Literature Search

Databases of Pubmed, Embase and Web of Science were overall searched for potentially eligible articles up to the date of March 19th, 2018. The search terms were defined as follows: (“d-dimer”) AND (“intracerebral hemorrhage” OR “cerebral hemorrhage” OR “brain hemorrhage” OR “hemorrhagic stroke” OR “intracranial hemorrhage”). The reference lists of relevant reviews were identified, and manual searching was also used to extract additional articles.

### Data Extraction and Quality Assessment

Two reviewers independently extracted the data from studies selected in the form of standardized data collections. Disagreements were resolved by joint review or by consulting the original article. The following general characteristics of the included studies were extracted: first author, publication year, country, detection method for d-dimer, other data containing proportion of male subjects, d-dimer levels and mean age both in ICH patients and healthy controls. The developed guidelines of preferred reporting items for systematic reviews and meta-analyses (PRISMA) were followed in our study (10) (Supplementary Table 1). The quality of all eligible studies was assessed by Newcastle-Ottawa Scale (NOS) (11) (Supplementary Table 2). The maximum score for each study was 9 points: selection of the study was 4 points, comparability of the groups was 2 points, and ascertainment of outcomes was 3 points. Studies with 6 points or higher were considered as good quality, while those with 5 points or less were regarded as suboptimal quality.

### Statistical Analysis

In this meta-analysis, the software Review Manager 5.2 and STATA version 14.0 were used to perform the statistical analysis. Standard mean difference (SMD) with 95% confidence intervals (CI) was calculated by applying either a fixed effects model or a random effects model, in order to evaluate the differences in d-dimer levels as well as confounding factors including sex, age, high blood pressure (HBP), diabetes mellitus (DM), smoking, and alcohol between the ICH patients and the control group. The pooled effect size was assessed by utilizing the *Z* test. Further, the *I*^2^ test (25, 50, and 75% represented low, moderate, and high degrees of heterogeneity) was used to reflect the between-study heterogeneity (12, 13). A fixed effects model was adopted, in case of insignificant heterogeneity amongst studies (*I*^2^ test exhibited ≤ 50%; and *p* > 0.05). Elsewhere, the random effects model was applied to calculate the pooled effect estimates (12, 14). Subgroup analyses, with respect to continent, sample size of participants (≤ 50 or more) and age (< 65 or older), in relation to plasma d-dimer between ICH patients and controls were also conducted. The source of heterogeneity was explained in the test for subgroup differences when *I*^2^ test exhibited ≥ 50% and *p* ≤ 0.05. Meta-regression analyses on sex, age, HBP, DM, smoking, and alcohol were conducted to evaluate the effect of the confounding factors for d-dimer levels. Sensitivity analysis was carried out to examine whether the pooled risk estimates could be impacted by any single study included in the meta data set. Publication bias was assessed using Egger's test (15).

## Results

### Study Selection and Characteristics

**Figure 1:**
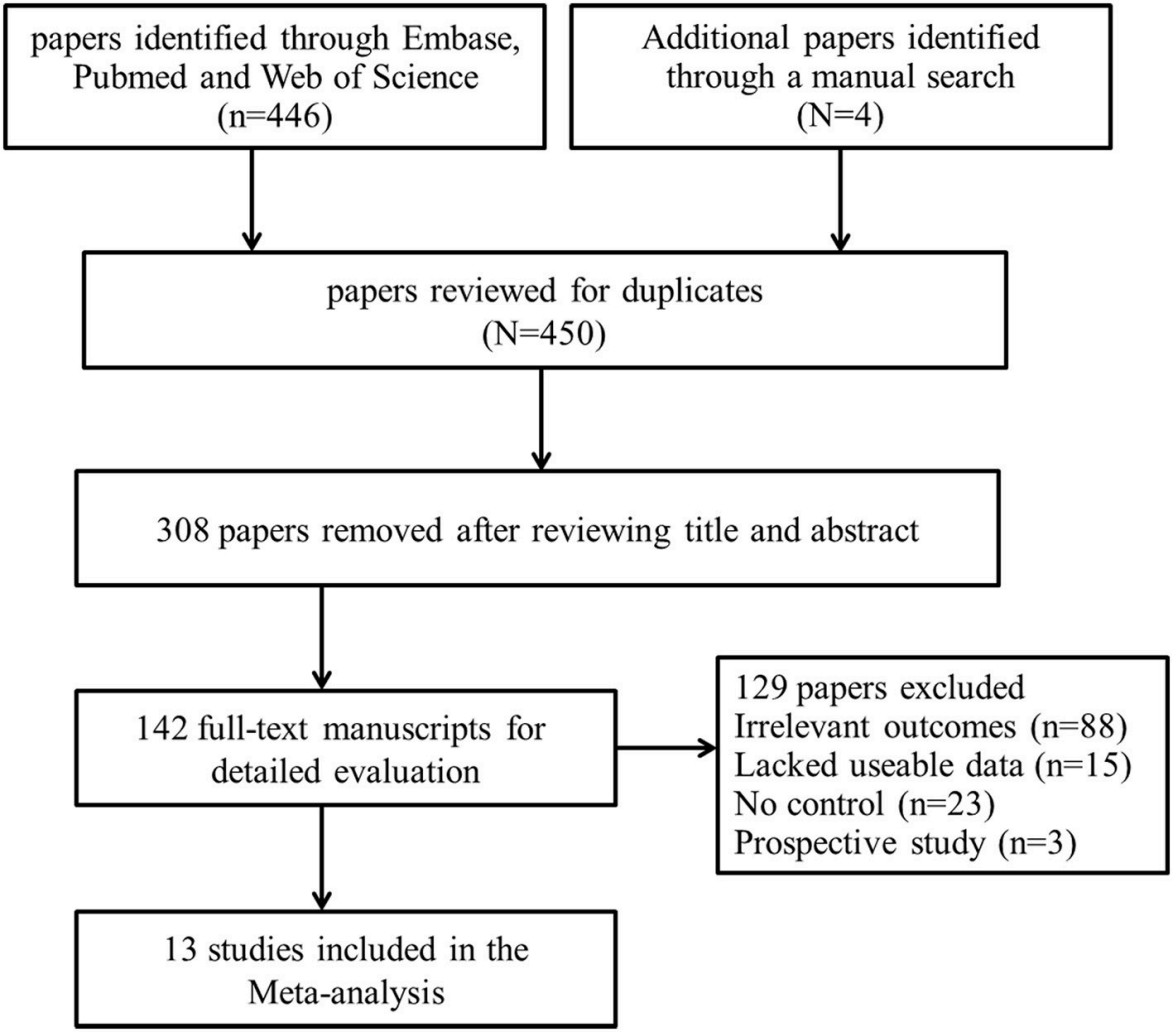
Flow chart of study selection in the meta-analysis.

**Figure 2:**
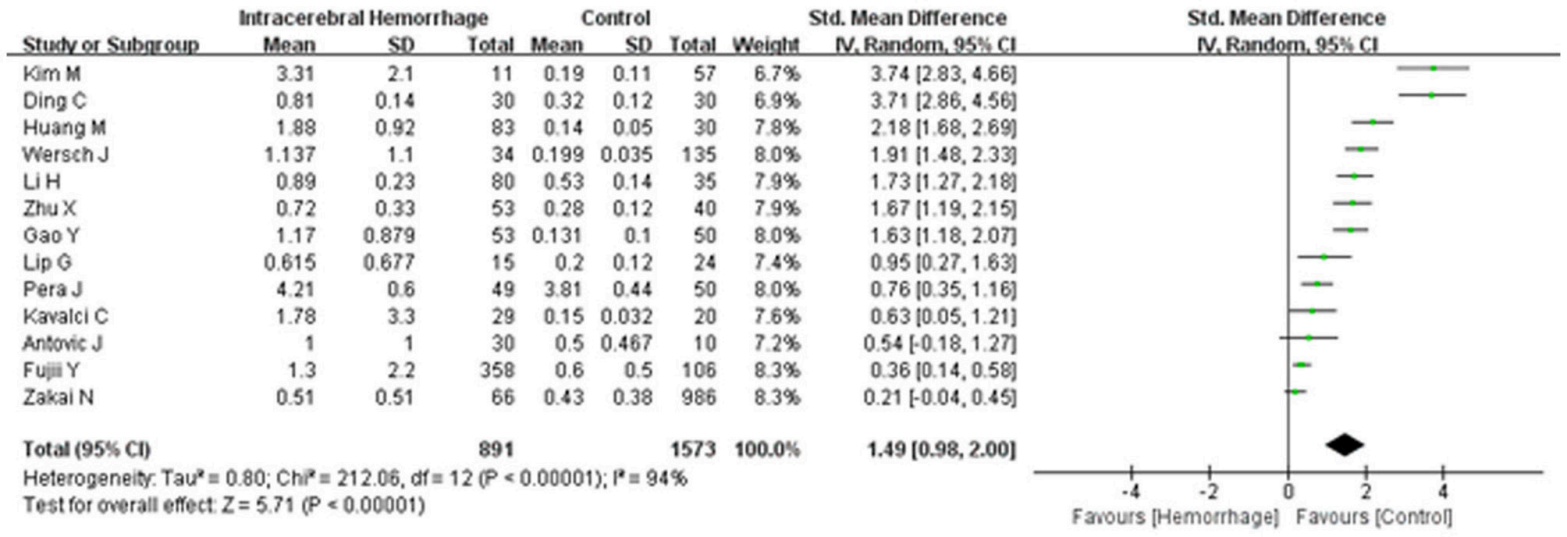
Forest plots for the comparisons of d-dimer levels between intracerebral hemorrhage (ICH) patients and healthy controls. CI, confidence interval.

**Figure 3:**
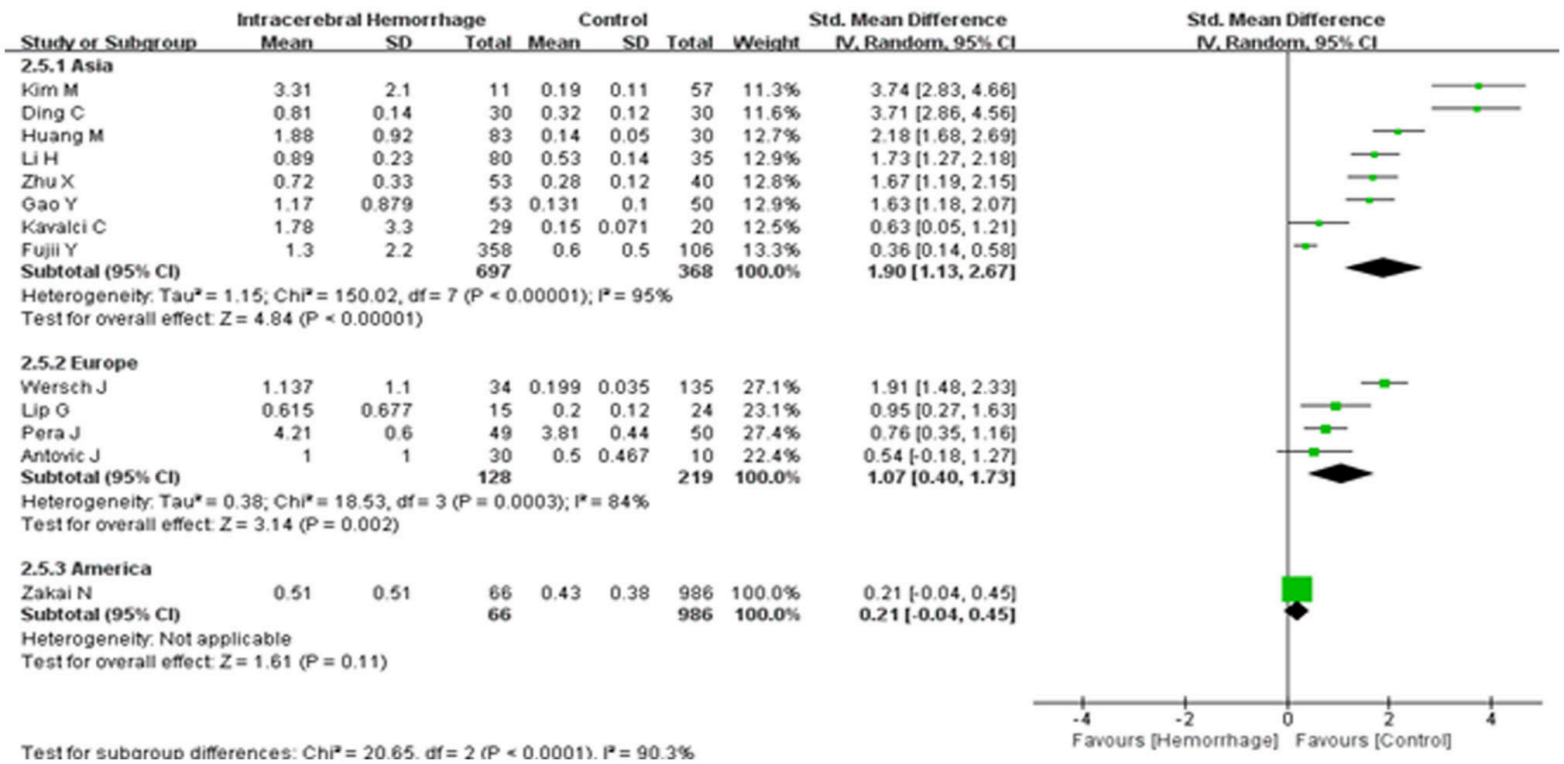
Forest plots of the subgroup analyses on continent in relation to d-dimer levels between intracerebral hemorrhage (ICH) patients and healthy controls. CI, confidence interval.

**Figure 4:**
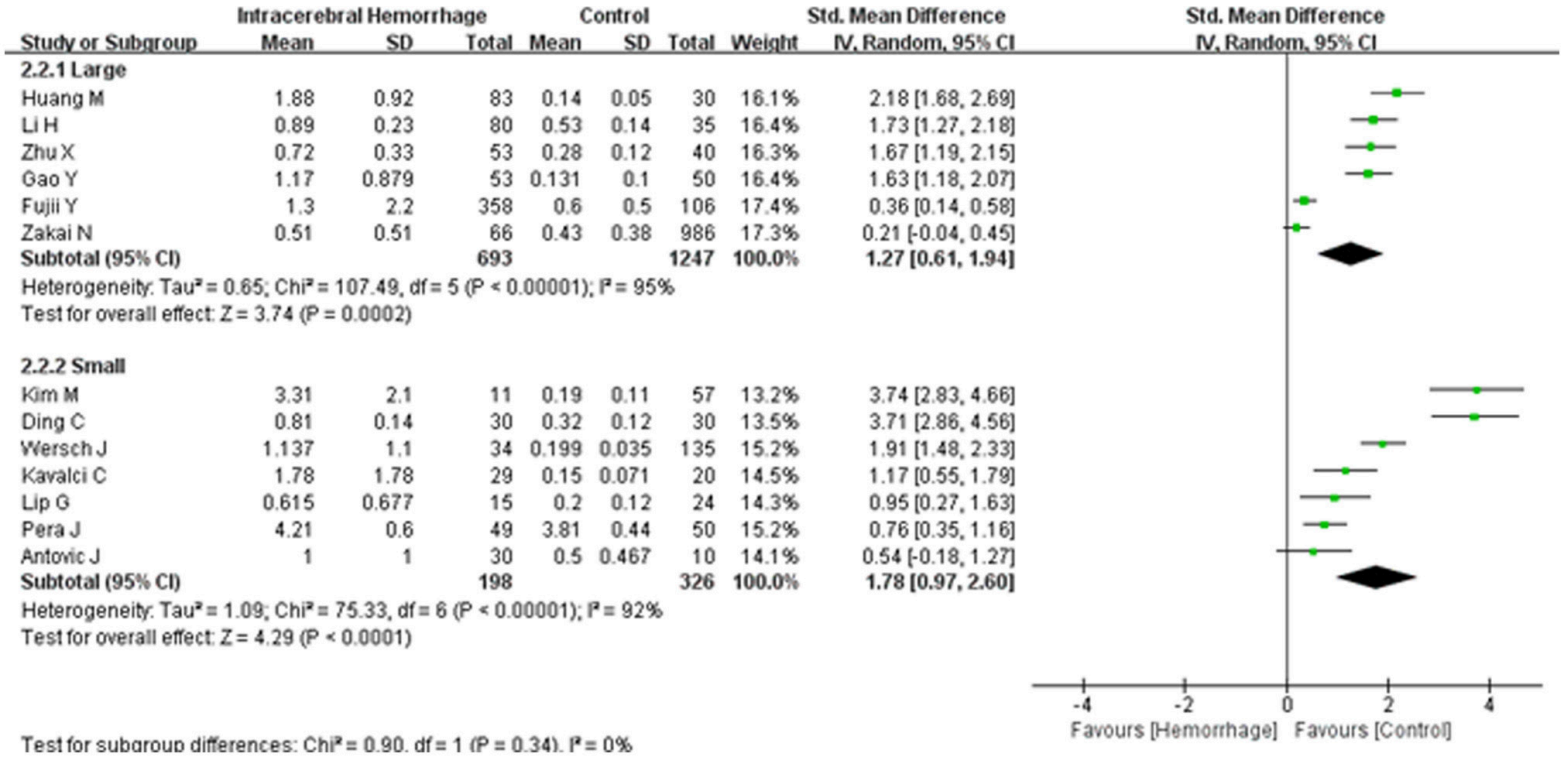
Forest plots of the subgroup analyses on sample size in relation to d-dimer levels between intracerebral hemorrhage (ICH) patients and healthy controls. CI, confidence interval.

**Figure 5:**
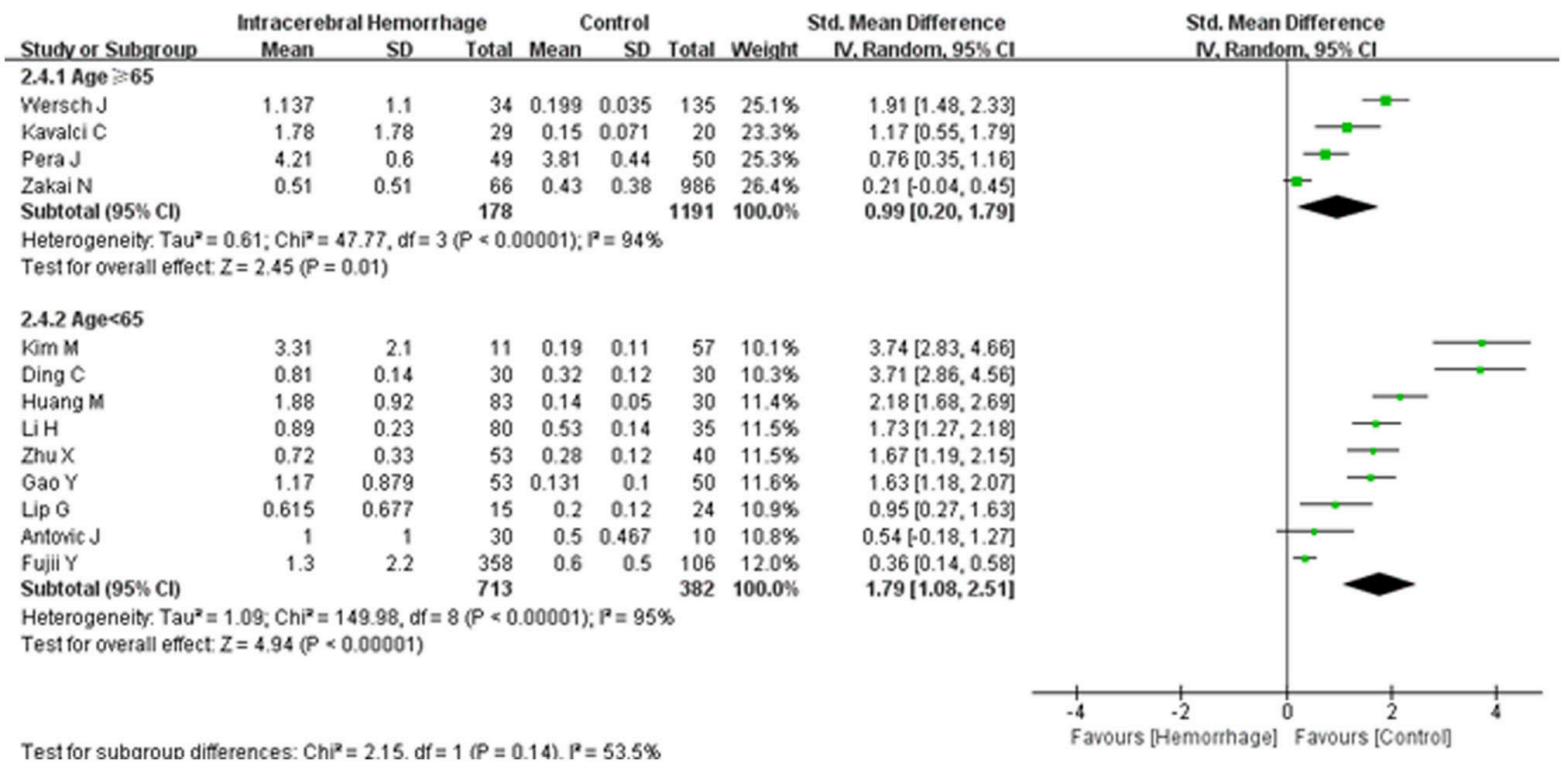
Forest plots of the subgroup analyses on age in relation to d-dimer levels between intracerebral hemorrhage (ICH) patients and healthy controls. CI, confidence interval.

Combining electronic database search with manual search, we initially retrieved 450 relevant titles and abstracts. Three hundred and eight duplicated or irrelevant studies were excluded after review of the abstracts. After screening the full text of the remaining 142 potentially relevant articles, 129 articles were further excluded, mainly including 88 articles with irrelevant outcomes, 15 articles that lacked usable data, 23 articles with no controls and 3 with prospective studies. Finally, 13 studies met our inclusion criteria and were included in our systematic review (5, 16–27) (Figure 1). Baseline characteristics of the 13 studies were summarized and all of them were categorized as good quality according to the NOS criteria (Table 1). A total of 2,464 participants, including 891 ICH patients and 1,573 healthy controls, were included in this meta-analysis. The d-dimer level data in ICH patients and healthy controls were extracted from the studies (Figure 2). Study subjects in eight of the thirteen researches were from Asia, with four studies conducted among Europe populations and the remaining one research was from America (Figure 3). The subgroup analysis on sample sizes, investigating d-dimer levels in ICH and healthy controls, showed that there were six studies with large sample cohorts (*n* > 50) and seven studies with small sample groups (*n* ≤ 50) (Figure 4). Subgroup analysis of age presented four studies with ICH patients over 65 years old and nine studies with ICH patients < 65 years old (Figure 5). The detailed flow diagram for literature inclusion and the screening steps are presented in Figure 1. The data of confounding factors including sex, age, HBP, hyperlipidemia, DM, smoking, alcohol, obesity, white blood cell, and hypersensitive C-reactive protein in ICH patients and healthy controls were extracted from the included studies (Supplementary Table 3). The meta-regression analyses were conducted on sex (*p* = 0.956, Adj *R*-squared = −15.12%), age (*p* = 0.469, Adj *R*-squared = −4.17%), HBP (*p* = 0.935, Adj R-squared = −26.06%), DM (*p* = 0.437, Adj R-squared = −7.65%), smoking (*p* = 0.287, Adj R-squared = 25.88%) and alcohol (*p* = 0.917, Adj R-squared = −102.34%) for the levels of d-dimer.

**Table 1:** General characteristics of the included studies.

**First author**	**Year**	**Country**	**Detecting methods**	**Hemorrhagic**			**Controls**			**Newcastle-Ottawa Scale**
				**No. of male patients**	**D-dimer, mg/L Mean ± SD**	**Age, Years**	**No. of male patients**	**D-dimer, mg/L Mean ± SD**	**Age, Years**	
Kavalci	2011	Turky	ELFA	14/29	1.78 ± 1.78	67.86 ± 12.6	8/20	0.15 ± 0.071	66.10 ± 8.6	8
Fujii	2001	Japan	ELISA	212/358	1.3 ± 2.2	< 65	NR/106	0.6 ± 0.5	Age-matched	8
Pera	2012	Poland	ITA	27/49	4.21 ± 0.6	69.6 ± 11.4	30/50	3.81 ± 0.44	65.9 ± 6.2	7
Lip	2001	England	ELISA	8/15	0.615 ± 0.677	64.2 ± 9.2	14/24	0.2 ± 0.12	65 ± 14	7
Wersch	1993	Netherlands	ELISA	20/34	1.137 ± 1.1	71.0 ± 11.6	NR/135	0.199 ± 0.035	55.3 (24–90)	7
Antovic	2002	Sweden	GICT	14/30	1 ± 1	63(33–74)	NR/10	0.5 ± 0.467	NR	6
Zakai	2017	America	ITA	41/66	0.51 ± 0.51	67 ± 9	481/986	0.43 ± 0.38	65 ± 3	6
Gao	2016	China	ELISA	31/53	1.17 ± 0.879	57.8 ± 16.2	27/50	0.131 ± 0.1	56.3 ± 15.5	8
Ding	2009	China	ITA	18/30	0.81 ± 0.14	58.12 ± 13.2	16/30	0.32 ± 0.12	56.15 ± 15.4	7
Huang	2009	China	ELISA	54/83	1.88 ± 0.92	60.9 ± 9.7	17/30	0.14 ± 0.05	62.9 ± 8.4	7
Zhu	2008	China	ELISA	30/53	0.72 ± 0.33	56.8 ± 15.2	20/40	0.28 ± 0.12	50.2 ± 12.8	6
Li	2007	China	ELISA	49/80	0.89 ± 0.23	61.7 ± 9.2	19/35	0.53 ± 0.14	62.3 ± 7.6	8
Kim	2010	Korea	ELFA	5/11	3.31 ± 2.10	56.4 ± 14.5	25/57	0.19 ± 0.11	43.8 ± 12.0	6

*M, male; NO, number; NR, not reported; ELFA, enzyme-linked fluorescence immunoassays; ELISA, enzyme-linked immunosorbent assay; ITA, immunoturbidimetric assay; GICT, Immune colloidal gold technique; SD, standard deviation*.

### Meta-Analysis

A random effects model was applied, as there was high heterogeneity (*I*^2^ = 94%) amongst the 13 studies included. The results of this meta-analysis revealed that higher levels of d-dimer were displayed in ICH patients than those observed in the healthy controls (95% CI = 0.98–2.00, *p* < 0.001) (Figure 2). Subgroup analysis on continent demonstrated that higher d-dimer levels were exhibited in ICH patients compared with healthy controls both in Asia (95% CI = 1.13–2.67, *p* < 0.001) and Europe (95% CI = 0.40–1.73, *p* < 0.001), whereas no significant differences were found in the comparisons of d-dimer levels between ICH group and controls in America (95% CI = −0.04–0.45, *p* < 0.001); and that this difference was statistically significant in the individual population subgroup comparisons (Test for subgroup differences: *I*^2^ = 90.3%, and *p* < 0.001) (Figure 3). Further subgroup analysis on either sample size (large samples size: 95% CI = 0.61–1.94, *p* < 0.001; small samples: 95% CI = 0.97–2.60, *p* < 0.001; Test for subgroup differences: *p* = 0.34) (Figure 4) or age (mean age ≥ 65 years: 95% CI = 0.20–1.79, *p* < 0.001; mean age < 65 years: 95% CI = 1.08–2.51, *p* < 0.001; Test for subgroup differences: *p* = 0.14) (Figure 5) showed that patients with ICH had statistically marginal higher levels of d-dimer in ICH patients than that of healthy controls; and no statistic differences were found in the subgroup comparisons. The odds ratios of confounding factors showed that age and HBP are positively associated with the risk of ICH (Supplementary Figure 1); while other parameters including sex, DM, smoking, and alcohol showed no statistically significant differences between ICH patients and healthy controls (Supplementary Figure 2).

### Sensitivity Analysis and Publication Bias

**Figure 6:**
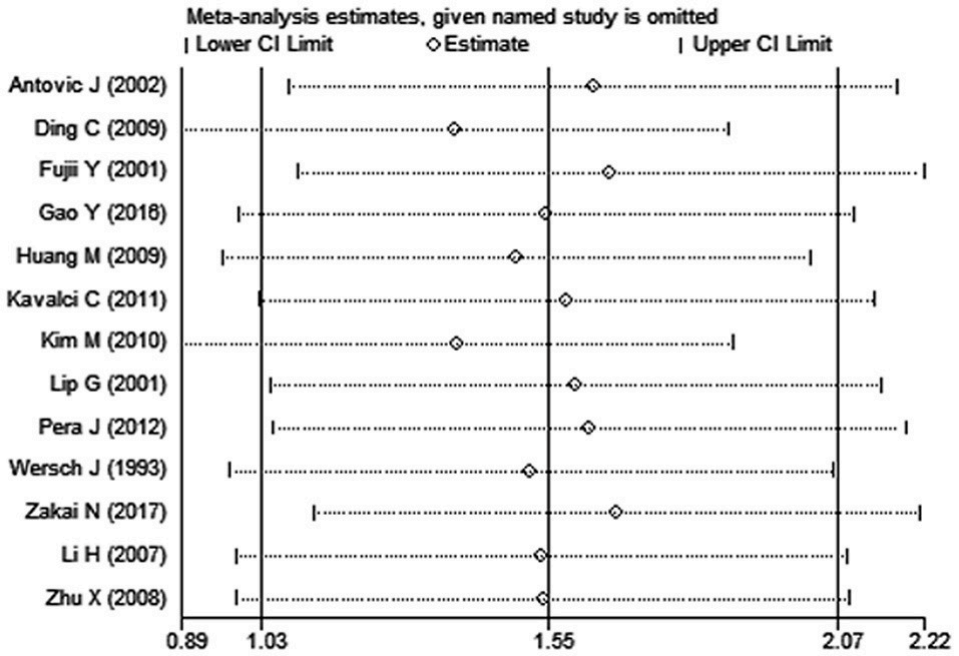
The plot in the sensitivity analysis of present meta-analysis (given named study was omitted).

**Figure 7:**
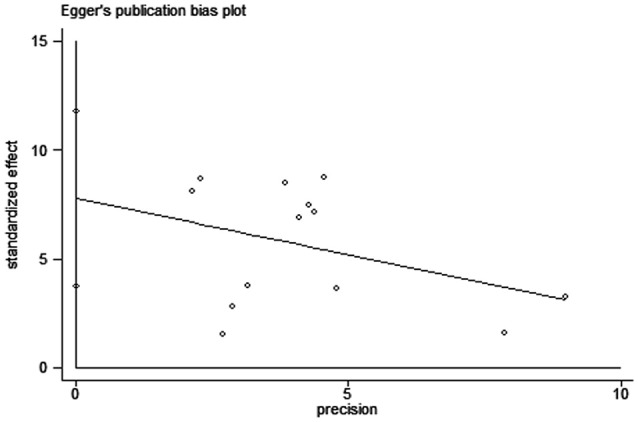
Egger's funnel plot to detect risk of publication bias in the meta-analysis. SMD: standard mean difference.

Sensitivity analysis performed by the leave-one-out approach showed that no single study had a substantial contribution to the pooled mean difference (Figure 6). The publication bias was evaluated by Egger's test (*p* = 0.01), the *P*-values of which indicated the probability of publication bias in the meta-analysis (Figure 7).

## Discussion

In current systematic overview, we summarized the studies of the existing evidence which passed our selection criteria to explore whether elevated levels of d-dimer would be associated with the risk of ICH. So far as we know, there is no similar meta-analysis including so many articles to investigate the potential linkage between them. A previous meta-analysis in which subgroup analysis contained as few as three articles to assess the hazard ratio of d-dimer for hemorrhagic stroke showed no causal relationship (28). On the contrary, a prospective study followed up for 9 years revealed that elevated d-dimer level appeared to be positively associated not only with ischemic stroke but also with hemorrhagic stroke (29). In view of the conflicting results from previous studies, it is possible to undertake an accurate estimate of the influence of plasma d-dimer on the risk of ICH by a larger comprehensive meta-analysis.

The results emerging from this meta-analysis revealed that plasma d-dimer levels of ICH patients were prominently higher than those of healthy controls, which implied the predictive role of d-dimer on the risk of ICH. To illustrate this point, previous evidence has supported that plasma d-dimer, as an indicator of fibrin turnover, reflects intricate disorders of fibrinolytic and hemostatic functions that may be related to different types of stroke (30, 31). In ischemic stroke and cardiovascular disease, the tendency to form systemic fibrin and thrombosis is more obvious with the increase of d-dimer level (32, 33). However, high d-dimer levels promote subclinical elevation of fibrinolytic function with subsequent plasmin generation in microvascular lesions of ICH, which might be responsible for the inhibition of hemostatic function and hypocoagulable state of the blood system, and thus easy to trigger massive hemorrhage (29). With the deterioration of coagulopathies, high level of d-dimer is more susceptible to the risk of ICH, whilst ICH patients with high d-dimer level are more prone to develop serious complications including mostly deep venous thrombosis, transient cardiomyopathies and disseminated intravascular coagulation (34, 35). On the other hand, elevated d-dimer level has reported to be closely related to large hematoma volume, the presence of intraventricular and subarachnoid extension, early mortality and poor functional recovery in ICH patients (35, 36). According to previous study involved retrospective analysis of prospectively collected data, d-dimer level > 1.9 mg/L and ICH volume >30ml emerged as independent indicators of early neurologic deterioration and mortality (36). Therefore, it is speculated that d-dimer lowering therapy might not only reduce the occurrence of ICH, but would also prevent the progression, reduce early mortality and improve functional outcomes of ICH.

However, we are practically unable to determine whether the level of d-dimer has already gone up before the onset of ICH as all blood samples of d-dimer were obtained on admission. A prospective study on recurrent stroke revealed that there were no significantly differences of d-dimer levels in patients with a history of ICH who would develop rehemorrhage at follow-up compared to ICH controls who did not go on to become cases, indicating that ICH event may exert no effect on the alterations of d-dimer levels (37). During the progression of ICH, the acute response of ICH event does not activate the hemostatic systems unless intraventricular hemorrhage or intracerebral bleeding expands into the ventricles, causing arterial blood in contact with cerebrospinal fluid directly, which results in the generation of thrombin-antithrombin complex and plasmin-antiplasmin complex, as well as d-dimer accumulation (5). In addition, there are probably other factors influencing d-dimer levels in ICH patients in our study. There is convincing evidence that plasma d-dimer levels were significantly increased in patients with cerebral AVM, which is considered as an important class of lesions for ICH (38). Previous study showed that plasma d-dimer is a useful auxiliary tool for detecting minor intracranial AVMs with local fibrin deposition as small nidus of AVM is frequently observed alongside lesions where hemorrhage occurs (38–40). Nevertheless, a large AVM nidus appears to predict more additional ICH events during follow-up than a small nidus (39). The pathological changes of AVM vessels are mainly manifested as dysplasia of internal elastic lamina, arterialization of dilated veins and intravascular microaneurysms, which account for the easy rupture of AVM and a cause of AVM-related ICH (41). Moreover, the angioarchitectural characteristics of specific slow-flow histopathological lesions such as draining veins, the proliferation of type-I and type-III collagen may cause blood stagnation and coagulation disorders in AVM patients, which result in the elevation of plasma d-dimer levels (42). It is possible to believe that there were some ICH patients with intraventricular hemorrhage and AVMs recruited in the present meta-analysis, which may be partly responsible for the outcomes that the levels of plasma d-dimer in ICH patients were higher than those in healthy controls.

Curiously, subgroup analysis on continent confirmed that higher d-dimer levels were exhibited in ICH patients compared to healthy controls in both Asia and Europe, whereas these alterations were not found in American patients with ICH. These inconsistent outcomes of d-dimer levels on different continents are probably associated with heritability, lifestyles, dietary structure, and environment. Further subgroup analysis based on either sample size or age revealed that higher levels of d-dimer were present in ICH patients than that of healthy controls, which indicated that neither sample size-related differences nor age-related diversities exert an effect on the overall results. Substantial heterogeneity was found among studies, which is partly due to region-specific differences as the value of *I*^2^ was 90.3% and *p* < 0.01 in the test for subgroup differences on continent. Other possible sources of clinical heterogeneity may be derived from gender distinction, measurement methods, acquisition time of blood samples, and comorbidities. Although the odds ratios of confounding factors showed that age and HBP were positively associated with the risk of ICH, it should be noted that these data were obtained from a limited number of studies that meet our inclusion criteria, which might lead to biased outcomes. Some confounding factors such as hyperlipidemia, obesity, white blood cell and hypersensitive C-reactive protein were not able to be analyzed because of the small number of studies included. Further meta-regression analysis revealed that all of the analyzed confounding factors including age, HBP, sex, DM, smoking, and alcohol exert no effect on the outcome of the relationship between plasma d-dimer and the risk of ICH. Although publication bias exists in the meta-analysis, the sensitivities of both Begg's funnel plot and Egger's test were poor due to < 20 included studies (43). The provenience of publication bias may be that the unpublished studies with negative outcomes and non-english articles were not included, as well as existing high heterogeneity amongst the studies.

### Limitations

Several limitations should be acknowledged in this meta-analysis as fellow. There are deficiencies in the design of included studies and meta-analysis, such as selection bias, confounding bias, recall bias, and reporting bias. Although meta-regression of multiple comorbidities showed on effect for d-dimer, other confounding factors such as regional differences, cut-off values of d-dimer and testing methods may impact the sensitivity and heterogeneity of the results. Another concern was that the proportion of ICH patients with intraventricular hemorrhage and AVMs recruited in the studies may partially affect the summary outcomes of d-dimer levels on the relative risk of ICH. The susceptibility to ICH associated with d-dimer is likely overestimated due to the presence of publication bias.

## Conclusion

Our meta-analysis provides strong evidence that elevated d-dimer level is positively associated with the risk of ICH, which supports that plasma d-dimer may be a potential risk factor for ICH, particularly in Asian and European patients with ICH rather than in American patients. There is no evidence for any sample size-related differences and age-related diversities in relation to d-dimer on the risk of ICH. We recommend further studies on the role of lowering d-dimer levels to investigate its possibility of reducing ICH risk, with the expectation to provide a promising biological target for early diagnosis, effective treatment and improved neurological outcomes of ICH.

## Author Contributions

The study was conceived by ZZ and CZ. Papers were selected, and data were extracted by ZZ and YL. The data were analyzed and interpreted by XZ, KK, JX, and HQ. The first draft of the manuscript was written by ZZ, MZ, and CZ. All authors reviewed the manuscript and approved the final version.

### Conflict of Interest Statement

The authors declare that the research was conducted in the absence of any commercial or financial relationships that could be construed as a potential conflict of interest.

## Supplementary Material

Supplementary Figure 1 Forest plots for the comparisons of age **(A)** and HBP **(B)** between intracerebral hemorrhage (ICH) patients and healthy controls. CI, confidence interval.

Supplementary Figure 2 Forest plots for the comparisons of sex **(A)**, DM **(B)**, smoking **(C)**, and alcohol **(D)** between intracerebral hemorrhage (ICH) patients and healthy controls. CI, confidence interval.

Supplementary Table 1 The developed guidelines of preferred reporting items for systematic reviews and meta-analyses (PRISMA).

Supplementary Table 2 The Newcastle-ottawa quality assessment scale for case-control studies.

Supplementary Table 3 Characteristics of included studies of confounding factors.
