# Supplementary figures and images for: Plasma D-Dimer Concentrations and Risk of Intracerebral Hemorrhage: A Systematic Review and Meta-Analysis
Source: Front Neurol. 2018 Dec 20;9:1114. doi: 10.3389/fneur.2018.01114 (PMC6306414; doi:10.3389/fneur.2018.01114)

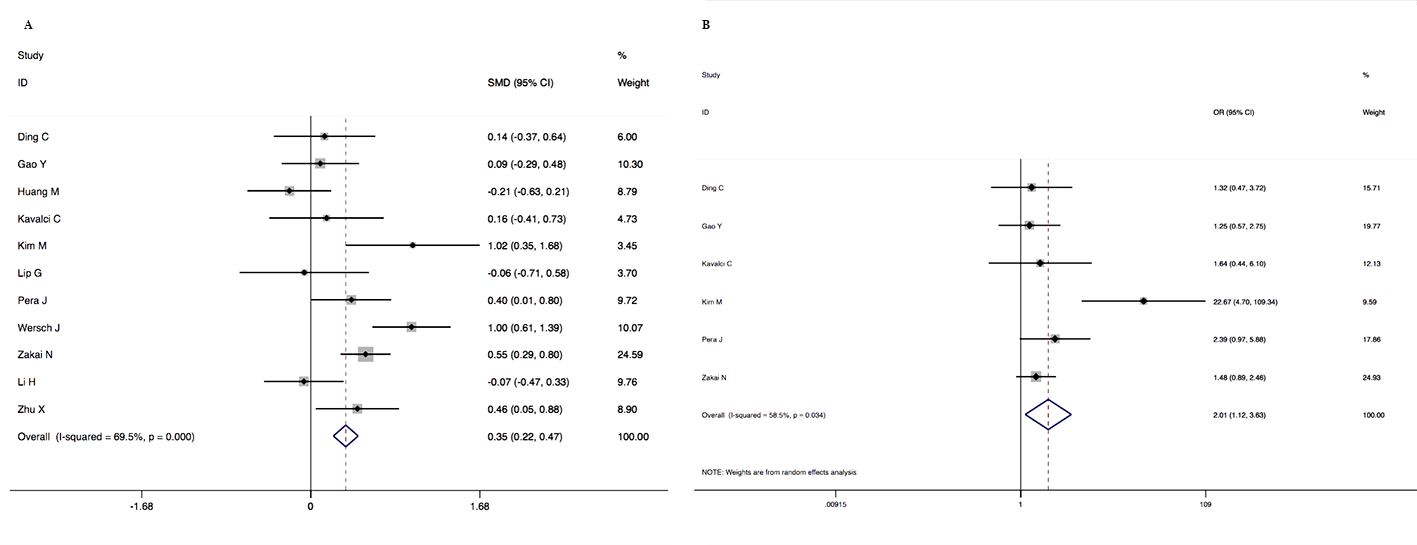

Supplement: Supplementary Figure 1 — Forest plots for the comparisons of age (A) and HBP (B) between intracerebral hemorrhage (ICH) patients and healthy controls. CI, confidence interval. [file Image_1.TIF]

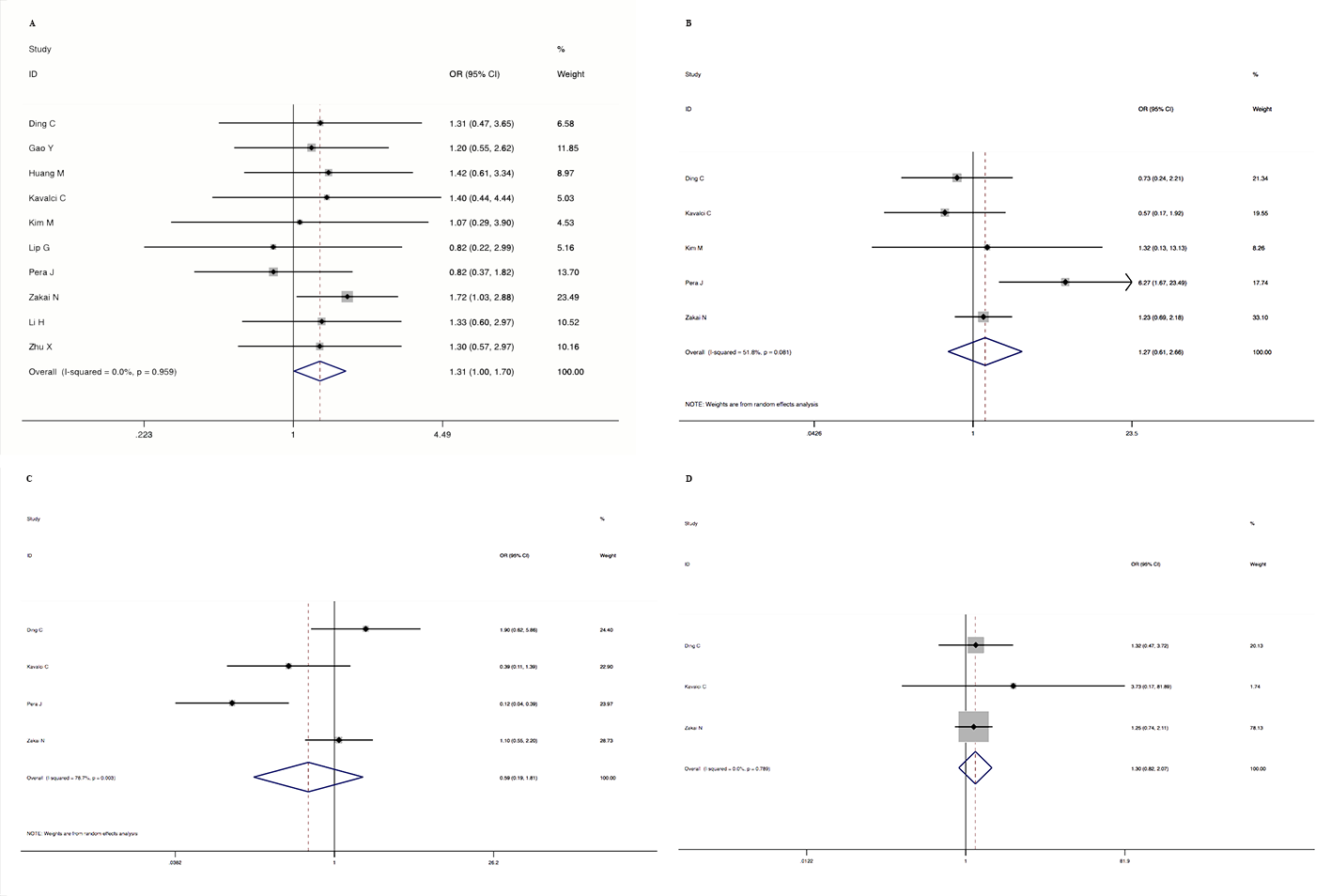

Supplement: Supplementary Figure 2 — Forest plots for the comparisons of sex (A), DM (B), smoking (C), and alcohol (D) between intracerebral hemorrhage (ICH) patients and healthy controls. CI, confidence interval. [file Image_2.TIF]
