# Supplementary material for: Plasma D-Dimer Concentrations and Risk of Intracerebral Hemorrhage: A Systematic Review and Meta-Analysis
Source: Front Neurol. 2018 Dec 20;9:1114. doi: 10.3389/fneur.2018.01114 (PMC6306414; doi:10.3389/fneur.2018.01114)
Supplement: Supplementary Table 2 — The Newcastle-ottawa quality assessment scale for case-control studies. [file Table_2.docx]

**The Newcastle-Ottawa Quality Assessment Scale for case-control studies**

| Study | Selection | Comparability | Outcome | Summary |
| --- | --- | --- | --- | --- |
| Kavalci C | 4 | 2 | 2 | 8 |
| Fujii Y | 4 | 2 | 2 | 8 |
| Pera J | 3 | 2 | 2 | 7 |
| Lip G | 3 | 2 | 2 | 7 |
| Wersch J | 3 | 2 | 2 | 7 |
| Antovic J | 3 | 1 | 2 | 6 |
| Zakai N | 3 | 1 | 2 | 6 |
| Gao Y | 4 | 2 | 2 | 8 |
| Ding C | 3 | 2 | 2 | 7 |
| Huang M | 3 | 2 | 2 | 7 |
| Zhu X | 4 | 0 | 2 | 6 |
| Li H | 4 | 2 | 2 | 8 |
| Kim M | 3 | 1 | 2 | 6 |
