# Supplementary material for: Plasma D-Dimer Concentrations and Risk of Intracerebral Hemorrhage: A Systematic Review and Meta-Analysis
Source: Front Neurol. 2018 Dec 20;9:1114. doi: 10.3389/fneur.2018.01114 (PMC6306414; doi:10.3389/fneur.2018.01114)
Supplement: Supplementary Table 3 — Characteristics of included studies of confounding factors. [file Table_3.DOCX]

| c-hsCRP_sd |  |  |  | 3.91 |  |  |  |  |  |  |  |  |  |  |
| --- | --- | --- | --- | --- | --- | --- | --- | --- | --- | --- | --- | --- | --- | --- |
| c-hsCRP_mean (mg/L) |  |  |  | 5.94 |  |  |  |  |  |  |  |  |  |  |
| t-hsCRP_sd |  |  |  | 6.32 |  |  |  |  |  |  |  |  |  |  |
| t-hsCRP_mean (mg/L) |  |  |  | 16.45 |  |  |  |  |  |  |  |  |  |  |
| c-WBC_sd |  |  | 1.6 |  |  |  |  |  |  |  |  |  |  |  |
| c-WBC_mean (×10^3^/mm^3^) |  |  | 6.7 |  |  |  |  |  |  |  |  |  |  |  |
| t-WBC_sd |  |  | 4.5 |  |  |  |  |  |  |  |  |  |  |  |
| t-WBC_mean(×10^3^/mm^3^) |  |  | 10 |  |  |  |  |  |  |  |  |  |  |  |
| cn-Obesity |  |  |  |  |  |  |  |  | 46 |  |  |  |  |  |
| c-Obesity |  |  |  |  |  |  |  |  | 4 |  |  |  |  |  |
| tn-Obesity |  |  |  |  |  |  |  |  | 34 |  |  |  |  |  |
| t-Obesity |  |  |  |  |  |  |  |  | 15 |  |  |  |  |  |
| cn-Alcohol |  | 13 |  |  |  | 20 |  |  |  |  | 690 |  |  |  |
| c-Alcohol |  | 17 |  |  |  | 0 |  |  |  |  | 296 |  |  |  |
| tn-Alcohol |  | 11 |  |  |  | 27 |  |  |  |  | 43 |  |  |  |
| t-Alcohol |  | 19 |  |  |  | 2 |  |  |  |  | 23 |  |  |  |
| cn-Smoking |  | 11 |  |  |  | 12 |  |  | 29 |  | 848 |  |  |  |
| c-Smoking |  | 19 |  |  |  | 8 |  |  | 21 |  | 138 |  |  | WBC whit blood cell, hsCRP hypersensitive C-reactive protein |
| tn-Smoking |  | 7 |  |  |  | 23 |  |  | 45 |  | 56 |  |  |  |
| t-Smoking |  | 23 |  |  |  | 6 |  |  | 4 |  | 10 |  |  |  |
| c-HL_sd |  |  |  |  |  |  |  | 1.6 | 1.14 |  |  |  |  |  |
| c-HL_mean (mmol/L) |  |  |  |  |  |  |  | 5.8 | 5.31 |  |  |  |  |  |
| t-HL_sd |  |  |  |  |  |  |  | 1.5 | 1.37 |  |  |  |  |  |
| t-HL_mean (mmol/L) |  |  |  |  |  |  |  | 6.9 | 5.27 |  |  |  |  |  |
| cn-DM |  | 8 |  |  |  | 12 | 53 |  | 47 |  | 769 |  |  |  |
| c-DM |  | 22 |  |  |  | 8 | 4 |  | 3 |  | 217 |  |  |  |
| tn-DM |  | 10 |  |  |  | 21 | 10 |  | 35 |  | 49 |  |  |  |
| t-DM |  | 20 |  |  |  | 8 | 1 |  | 14 |  | 17 |  |  |  |
| cn-HBP (mmHg) |  | 13 |  | 31 |  | 6 | 51 |  | 19 |  | 483 |  |  |  |
| c-HBP (mmHg) |  | 17 |  | 19 |  | 14 | 6 |  | 31 |  | 503 |  |  |  |
| tn-HBP (mmHg) |  | 11 |  | 30 |  | 6 | 3 |  | 10 |  | 26 |  |  |  |
| t-HBP (mmHg) |  | 19 |  | 23 |  | 23 | 8 |  | 39 |  | 40 |  |  |  |
| c-Age_sd |  | 15.4 |  | 15.5 | 8.4 | 8.6 | 12 | 14 | 6.2 | 16.5 | 3 | 7 | 12.8 |  |
| c-Age_mean(years) |  | 56.15 |  | 56.3 | 62.9 | 66.1 | 43.8 | 65 | 65.9 | 55.3 | 65 | 62.3 | 50.2 |  |
| t-Age_sd |  | 13.2 |  | 16.2 | 9.7 | 12.6 | 14.5 | 9.2 | 11.4 | 11.6 | 9 | 9.2 | 15.2 | t- test, c- control, tn- test no, cn- control no, smd std mean difference, sd standard deviation, HBP high blood pressure, DM diabetes mellitus, HL hyperlipemia, |
| t-Age_mean(years) |  | 58.12 |  | 57.8 | 60.9 | 67.86 | 56.4 | 64.2 | 69.6 | 71 | 67 | 61.7 | 56.8 |  |
| c-Femal |  | 14 |  | 23 | 13 | 12 | 32 | 10 | 20 |  | 505 | 34 | 20 |  |
| c-Male |  | 16 |  | 27 | 17 | 8 | 25 | 14 | 30 |  | 481 | 1 | 20 |  |
| t-Female | 16 | 12 | 146 | 22 | 29 | 15 | 6 | 7 | 22 | 14 | 25 | 31 | 23 |  |
| t-Male | 14 | 18 | 212 | 31 | 54 | 14 | 5 | 8 | 27 | 20 | 41 | 49 | 30 |  |
| _seES | 0.371 | 0.434 | 0.111 | 0.228 | 0.259 | 0.315 | 0.466 | 0.348 | 0.208 | 0.219 | 0.127 | 0.233 | 0.244 |  |
| _ES | 0.554 | 3.758 | 0.359 | 1.637 | 2.199 | 1.186 | 3.788 | 0.972 | 0.761 | 1.914 | 0.205 | 1.738 | 1.682 |  |
| smd | 3.74 | 3.71 | 2.18 | 1.91 | 1.73 | 1.67 | 1.63 | 0.95 | 0.76 | 0.63 | 0.54 | 0.36 | 0.21 |  |
| c-total | 30 | 30 | 106 | 50 | 30 | 20 | 57 | 24 | 50 | 135 | 986 | 35 | 40 |  |
| t-total | 30 | 30 | 358 | 53 | 83 | 29 | 11 | 15 | 49 | 34 | 66 | 80 | 53 |  |
| auther | Antovic J | Ding C | Fujii Y | Gao Y | Huang M | Kavalci C | Kim M | Lip G | Pera J | Wersch J | Zakai N | Li H | Zhu X |  |
